# Enhanced near-infrared absorber: two-step fabricated structured black silicon and its device application

**DOI:** 10.1186/s11671-018-2741-9

**Published:** 2018-10-10

**Authors:** Hao Zhong, Nasir Ilyas, Yuhao Song, Wei Li, Yadong Jiang

**Affiliations:** 1 State Key Laboratory of Electronic Thin Films and Integrated Devices, University of Electronic Science and Technology of China, Chengdu, 610054 China; 2 School of Optoelectronic Science and Engineering, University of Electronic Science and Technology of China, Chengdu, 610054 China

**Keywords:** Black silicon, Light absorptance, Band gap, Device responsivity

## Abstract

Silicon is widely used in semiconductor industry but has poor performance in near-infrared photoelectronic devices because of its high reflectance and band gap limit. In this study, two-step process, deep reactive ion etching (DRIE) method combined with plasma immersion ion implantation (PIII), are used to fabricate microstructured black silicon on the surface of C-Si. These improved surfaces doped with sulfur elements realize a narrower band gap and an enhancement of light absorptance, especially in the near-infrared range (800 to 2000 nm). Meanwhile, the maximum light absorptance increases significantly up to 83%. A Si-PIN photoelectronic detector with microstructured black silicon at the back surface exhibits remarkable device performance, leading to a responsivity of 0.53 A/W at 1060 nm. This novel microstructured black silicon, combining narrow band gap characteristic, could have a potential application in near-infrared photoelectronic detection.

## Background

Until now, many micro- and nanostructured black silicon materials can also be manufactured by using DRIE treatment and ion implantation, aiming to reduce light reflectance and enhance the near-infrared absorptance [1–5]. DRIE process is usually carried out in a mode of cyclic etching-passivation steps with a photoresist mask which can enable the silicon microfabrication of high-aspect ratio structures. Generally, this approach utilizes F-based plasmas such as SF_6_ for fast isotropic etching and then switches to a sidewall passivating cycle using C_4_F_8_ [6–8]. During the subsequent etching cycle, the passivating film is preferentially removed from the bottom of the groove due to ion bombardment, while preventing the etching of the sidewalls [9]. Finally, the alternating of etching and passivating cycles could form the specific geometries of the etched silicon structures, which depend mainly on mask size, gas flow, electrode power, process time, cycle times, and so on. In order to enhance the absorption of silicon in the near-infrared wavelength, the etched silicon structures will be doped by ion implantation after DRIE process. Under certain conditions, the black silicon arrays can be obtained, and the resulted sulfur dopants contained within the silicon lattice will eventually cause significant absorptance of below band gap radiation [10, 11].

As one of the most important material in semiconductor industry, black silicon has been widely used in sensitive photoelectronic detectors, solar cells, biochemical sensors, display devices, and optical communication objects [12–20]. Micro- and nanostructures of black silicon have been the focus of intense researches in recent years due to their extensive device application. A Si-PIN photoelectronic detector with black silicon at the front surface has been investigated in our early study [21]. This device structure has a wide depletion layer so that it can reduce the influence of carrier diffusion movement and achieve the purpose of improving device sensitivity and response speed. It is also noticed that using black silicon as a photosensitive surface is very difficult for the generated carriers to be collected by P layer to output photocurrent through electrode, resulting in a relatively low visible light response compared with a traditional Si-PIN detector. So, it appears a query that if a Si-PIN photoelectronic detector with black silicon at the back surface could complete two tasks at one time, i.e., to increase device sensitivity not only in the near-infrared but also in the visible wavelength?

In this article, we report the light absorptance enhancement and narrower band gap characteristic of microstructured black silicon fabricated by two-step process: DRIE combined with PIII. The effect of different etching process on the light absorptance in the wavelength range from 400 to 2000 nm have been studied, and the detector based on this microstructured black silicon at the back surface has also been investigated with an emphasis on device responsivity in the wavelength of 400~1100 nm.

## Methods

**Fig. 1:**
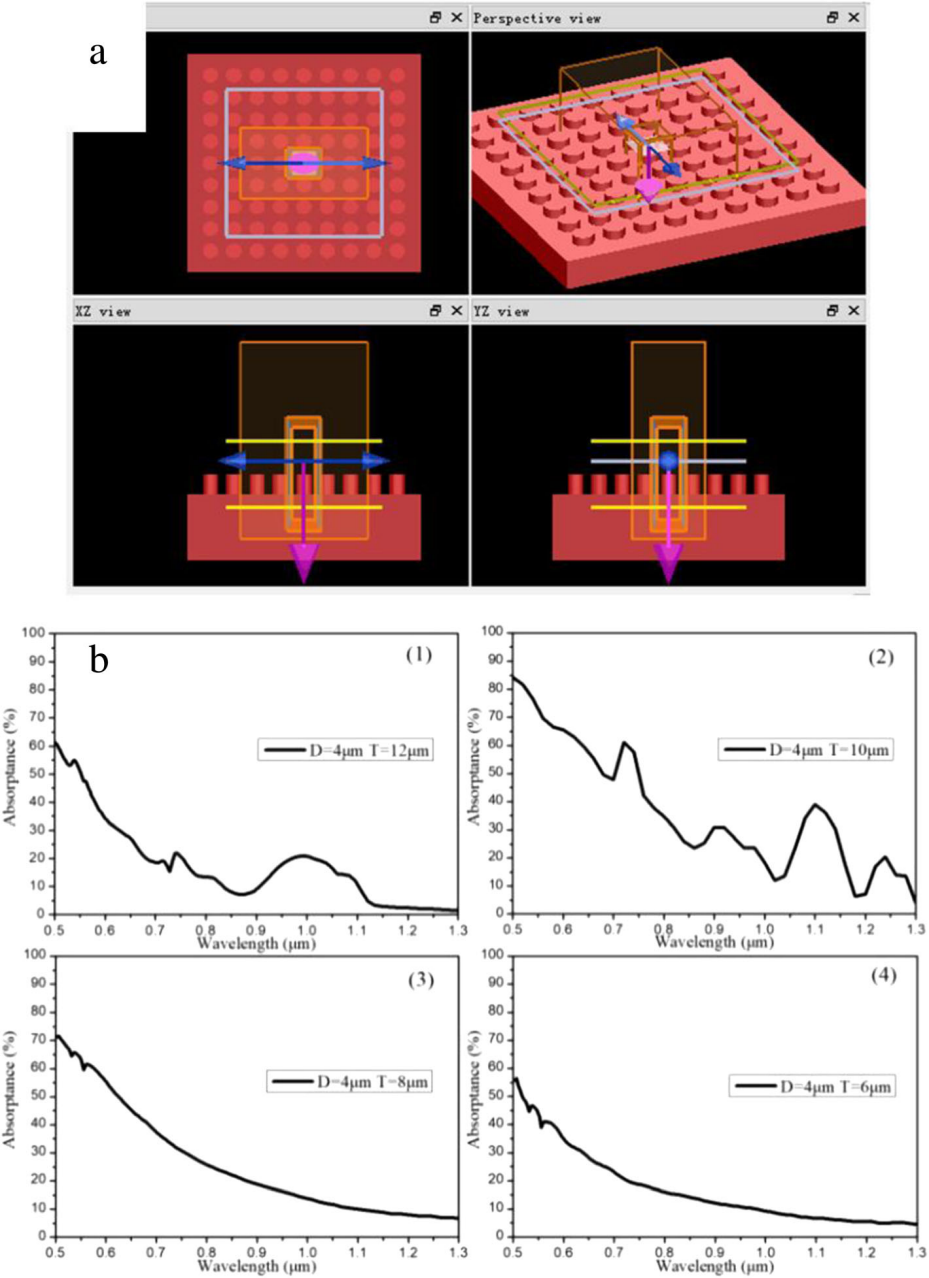
Optical simulation model (**a**) and simulation results (**b**) of microstructured silicon

As shown in Fig. 1a, uniform and periodic distributing cylindrical arrays were chosen to research the optical properties of microstructured silicon by FDTD Simulation software. Figure 1b represents the relationship between the absorbance and four different model sizes of the microstructured silicon after optimized simulation, in which four models have the same cylinder diameter (*D* = 4 μm) but different center distance (*T*_1_ = 12 μm, *T*_2_ = 10 μm, *T*_3_ = 8 μm, *T*_4_ = 6 μm).

**Fig. 2:**
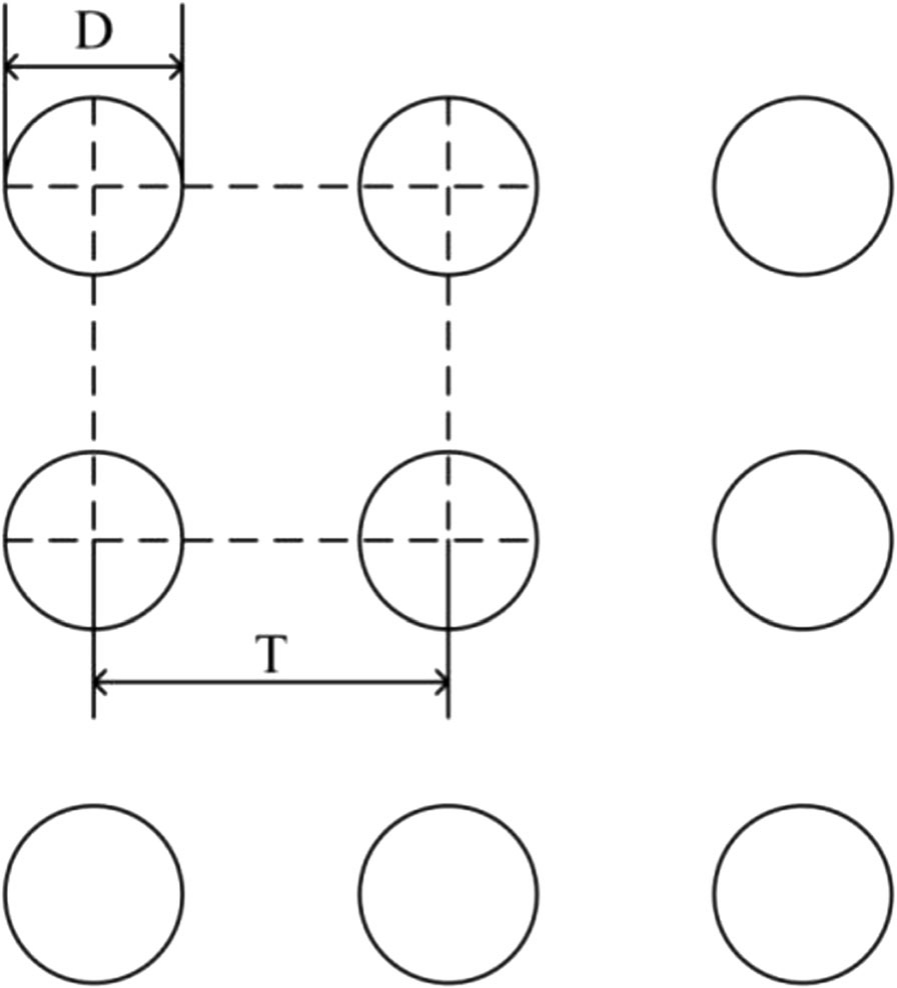
The schematic diagram of mask size

As shown in Fig. 2, based on the above optimized simulation results, three different photolithography mask were designed by varying the mask size named as mask I (*D* = 4 μm, *T* = 6 μm), mask II (*D* = 4 μm, *T* = 8 μm), and mask III (*D* = 4 μm, *T* = 10 μm), respectively. Then photoresist NR9-1500PY was applied to deposit circular arrays mask on the polishing surface of silicon pieces (15 × 15 cm^2^), which were cut from n-type silicon wafers with a thickness of 500 μm and a resistivity of 2500 to 3000 Ω · cm. In order to investigate the effect of etching process on light absorptance of microstructured silicon, we moved the testing samples resulted from mask III into process cavity (DRIE, ICP-100D) and changed the cycle times, in which SF_6_ was used as etching gas and C_4_F_8_ as passivating gas, for 30 times, 70 times, and 100 times, differently. After etching processes, the silicon pieces were removed of any residual photoresist under the atmosphere of oxygen to ensure that the silicon surface was clean and smooth. In order to enhance the absorptance of microstructured silicon, especially in near-infrared band, the testing samples resulted from mask III hereafter were doped with sulfur elements through PIII process under the condition of 1.0E + 15 cm^− 2^ implantation dose and 800 eV implantation energy, respectively.

The morphologies of black silicon were characterized by a field emission scanning electron microscope (SEM, JSM-7500F). The light absorptance was obtained at room temperature using a fiber optic spectrometer (NIR2500) equipped with an integrating sphere (Idea Optics, IS-20-5). The detector responsivity was measured by using an optical power meter (OPHIR, Vega), an optical chopper (Scitec Instruments, Model-300CD), and a Keithley2636B apparatus under the dark room environment. In order to ensure the accuracy of the measurement, we carried out calibration before test and each of these measurements was performed on a few samples (usually 4 to 6).

## Results and Discussion

**Fig. 3:**
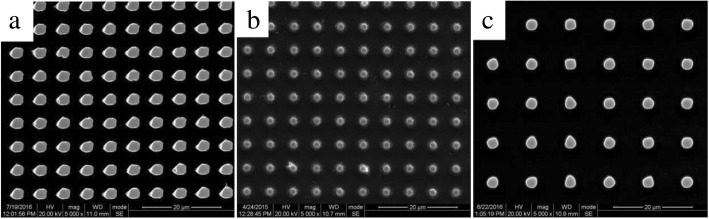
Typical microstructured silicon arrays made by DRIE for different mask sizes. **a** Mask I. **b** Mask II. **c** Mask III

Figure 3 gives the typical SEM images of aligned microstructured silicon arrays which are perpendicular to the surface of substrate for three different mask sizes. It is clearly shown that the top view of the microstructured silicon is not actually standard circles due to the fact that DRIE process mainly depends on the mask size and the quality of photolithography technique. Then, in order to investigate the effect of etching process on light absorptance of microstructured silicon, we change the cycle times as 30, 70, and 100 times on mask III testing samples under the conditions of etching with SF_6_ for 3 s, and passivating with C_4_F_8_ for 2 s, every cycle time.

**Fig. 4:**
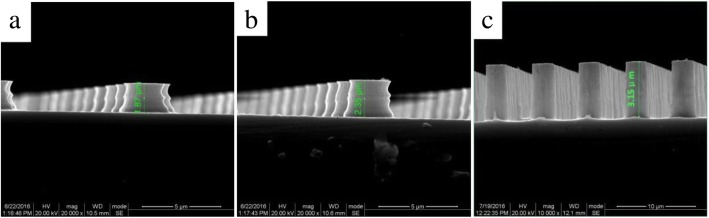
Sectional views of mask III samples fabricated for different cycle times. (**a**) 30, (**b**) 70, and (**c**) 100

According to the different etching rate ratio between photoresist and silicon, the etching depth can be controlled by process parameters as long as the photoresist is thick enough as a mask. It can be seen from Fig. 4 that the height of cylinders increases with the number of cycle times, in which the height from the top to the substrate are about 1.87 μm, 2.35 μm, and 3.15 μm, respectively. It is well known that in DRIE process, although there are passivating gases to protect the side wall of the etching target, it is still more or less accompanied by lateral etching. This is the reason why the resulting morphologies are not ideal cylindrical arrays. Obviously, the morphologies of these microstructured silicon arrays can be well controlled by varying lithography process and etching cycle times.

**Fig. 5:**
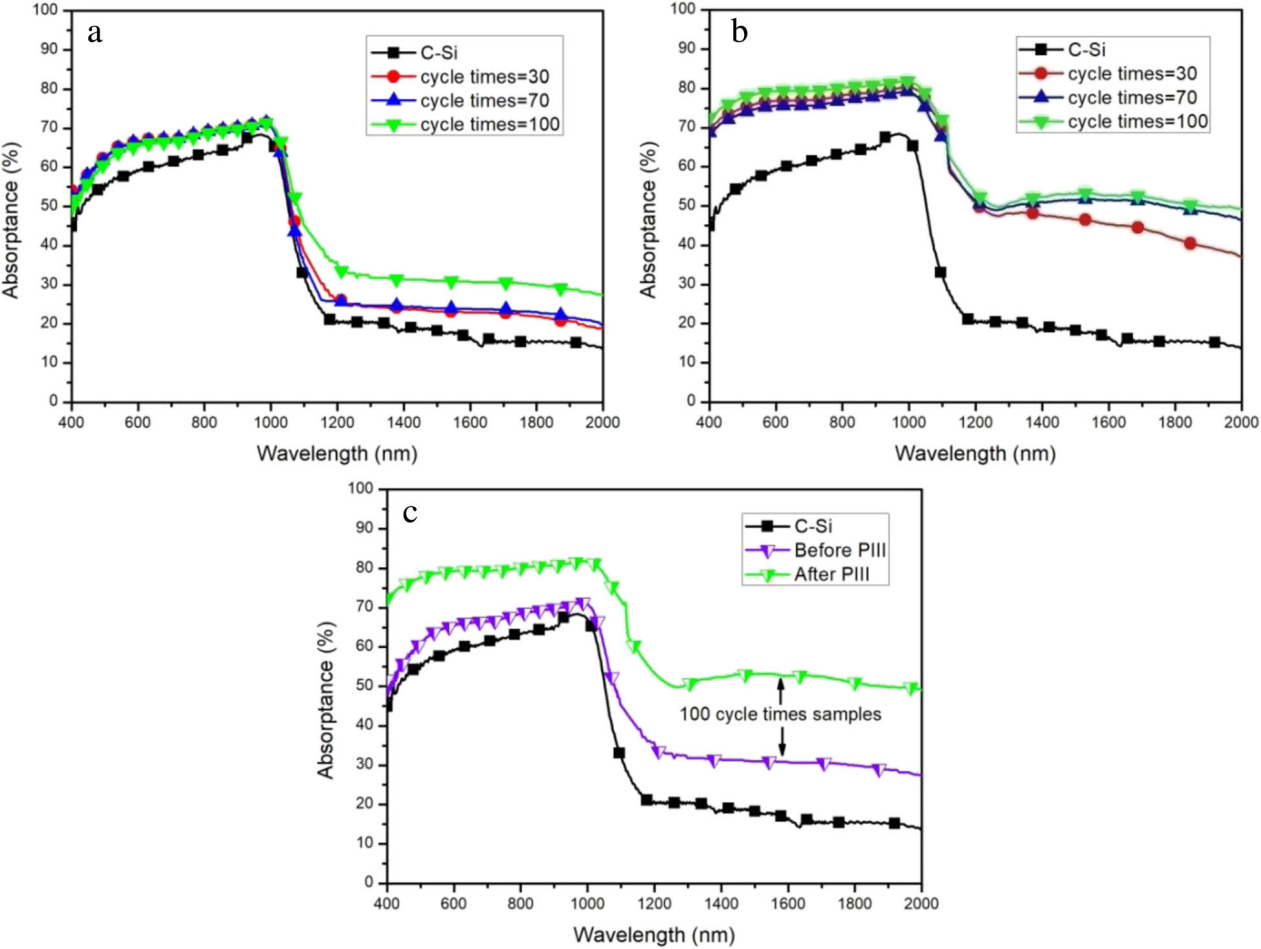
Absorptance of C-Si and black silicon fabricated by different cycle times before (**a**) and after PIII (**b**) and comparison of 100 cycle samples (**c**)

**Fig. 6:**
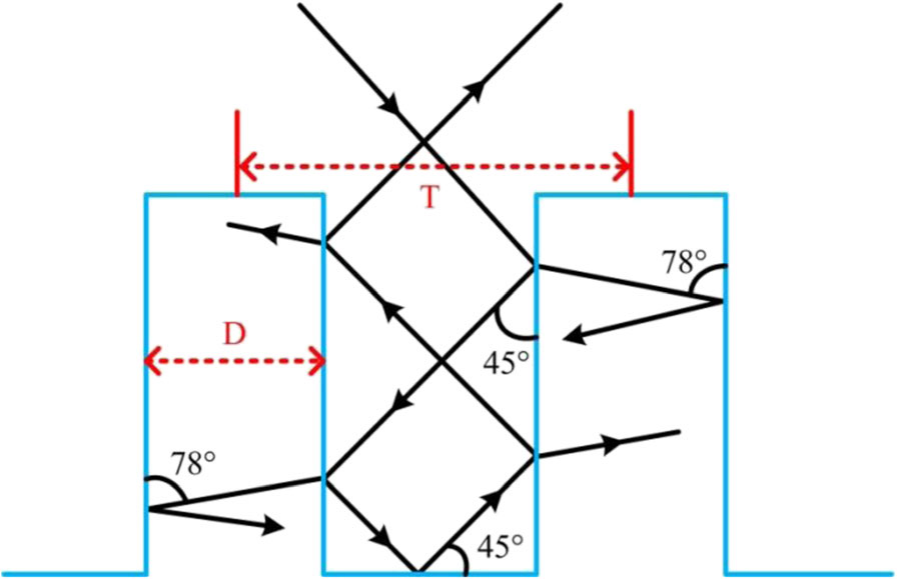
The transmission path of incident light on the surface of microstructured silicon

Figure 5a represents the light absorptance of microstructured cylindrical arrays at different cycle times without PIII process. It is shown that C-Si with etched silicon arrays, compared to ordinary C-Si, can enhance light absorptance throughout the wavelength range from 400 to 2000 nm to a certain extent. The sample etched for 100 cycle times presents the highest absorptance, up to 70% in the NIR range (800 to 2000 nm), and the average absorptance of this sample reaches 55% in the wavelength range from 400 to 2000 nm. This is due to the multiple reflection and absorption of microstructured silicon (as in Fig. 6). In the course of incident light being reflected continuously on the side surface of the cylinder, the absorption path length of incident light increases, resulting in the absorptance enhancement. Nevertheless, the absorption rate is still not high enough when the wavelength is more than 1000 nm. Therefore, in order to further improve the absorptance of microstructured silicon, especially in the near-infrared band, the same samples are doped with sulfur elements by PIII process under the condition of 1.0E + 15 cm^− 2^ implantation dose and 800 eV implantation energy, respectively. As shown in Fig. 5b, the light absorptance is obviously increased in the wavelength range from 400 to 2000 nm after ion implantation. Here, the light absorptance of the sample etched for 100 cycle times is much higher than that of C-Si. The maximum and average light absorptance increase significantly up to 83% and 62%, respectively. Moreover, one can easily observe (as in Fig. 5c) that the absorptance of 100 cycle samples has a significant change in the wavelength from 800 to 2000 nm before and after sulfur elements doping, in which the maximum and average values increase by 13% and 7%, respectively.

This high absorptance mainly comes from the sulfur doping among microstructured cylindrical arrays, forming multiple impurity levels in the energy band structure of C-Si. As a result, when these induced multiple impurity levels overlap, a new impurity band is formed after broadening, which finally reduces the band gap of C-Si. The band gap can be obtained from the absorptance spectrum of the sample by Tauc mapping. The specific steps adopted are as follows:

(i) the reflectance spectrum is converted to K-M function *F*(*R*_∞_) by using the Kubelka-Munk theory:

1 $$ F\left(R\infty \right)\approx \frac{A^2}{2R} $$

in which *R* and *A* are the reflectance spectrum and absorptance spectrum of the sample, respectively.

(ii) The K-M function *F*(*R*_∞_) is substituted into the Tauc formula as follows:

2 $$ {\left( hv\alpha \right)}^{1/n}=K\left( hv- Eg\right) $$

3 $$ hv=\frac{1239.7}{\lambda } $$

in which the value of index n is related to the transition type of the sample: direct transition, *n* = 1/2; indirect transition, *n* = 2. *F*(*R*_∞_) is proportional to the absorption coefficient α, which can be replaced by *F*(*R*_∞_), and *n* = 2 is substituted into the formula (2) to obtain:

4 $$ {\left( hv F\left(R\infty \right)\right)}^{1/2}=K\left( hv- Eg\right) $$

(iii) The reflectance and absorptance spectral data of the sample are substituted into Eq. (1), and Eq. (1) is substituted into Eq. (4), with *hv* as the abscissa (*X* axis) and (*hvF(R*_*∞*_))^1/2^ as the ordinate (*Y* axis).

(iv) The inflection point (the maximum point of the first derivative) is obtained by calculating the first derivative of the *hv*-(*hvF(R*_*∞*_))^1/2^ curve, and the tangent of the curve is made at this point. The abscissa value of the intersection of the tangent and the *X* axis are the band gap of the sample.

**Fig. 7:**
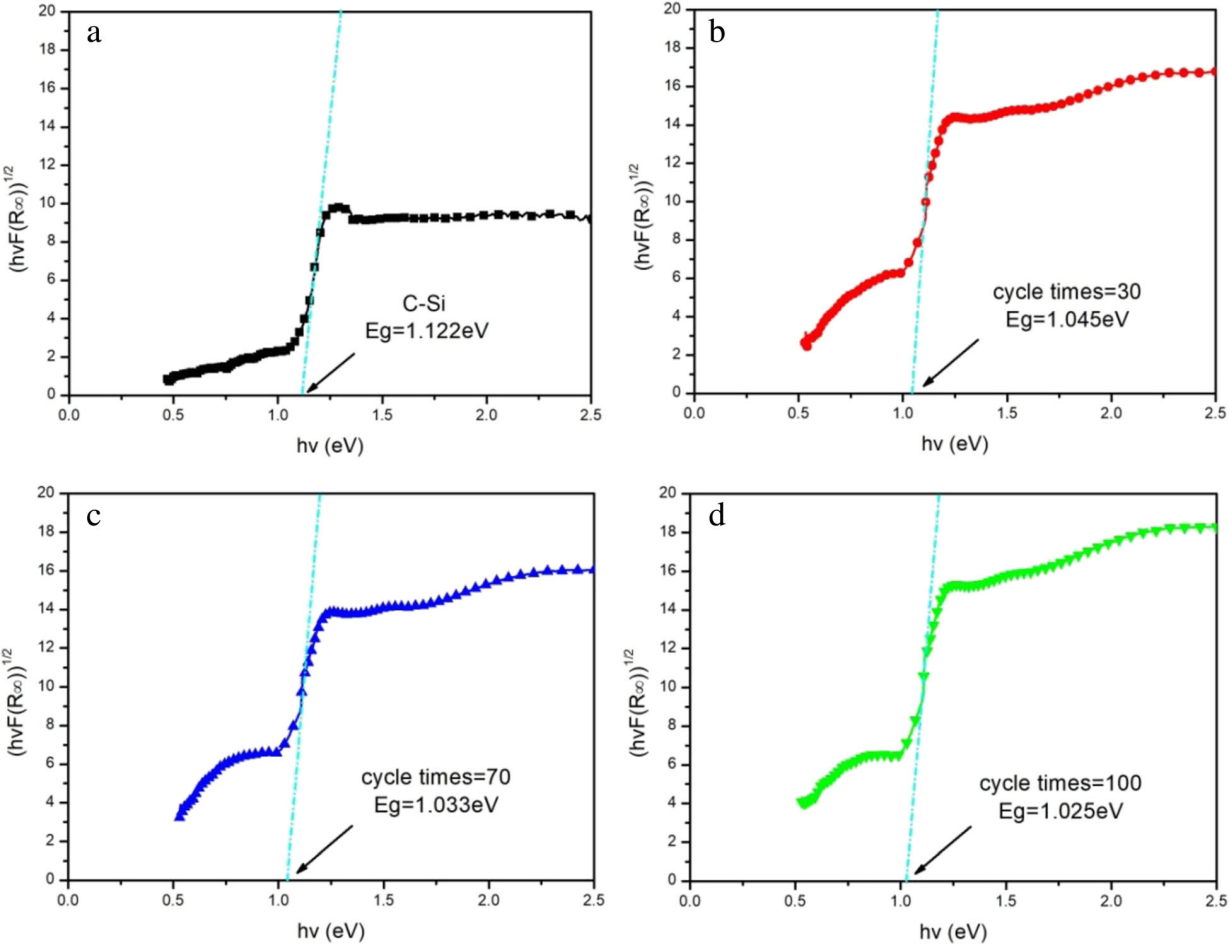
Band gaps of C-Si (**a**) and black silicon made by different cycle times: (**b**) 30, (**c**) 70, (**d**) 100

Figure 7 shows final calculated band gaps of C-Si and black silicon with different cycle times. It can easily be found that three lower band gaps of black silicon as 1.045 eV, 1.033 eV, and 1.025 eV are obviously decreased, respectively, compared to 1.12 eV band gap of C-Si.

**Fig. 8:**
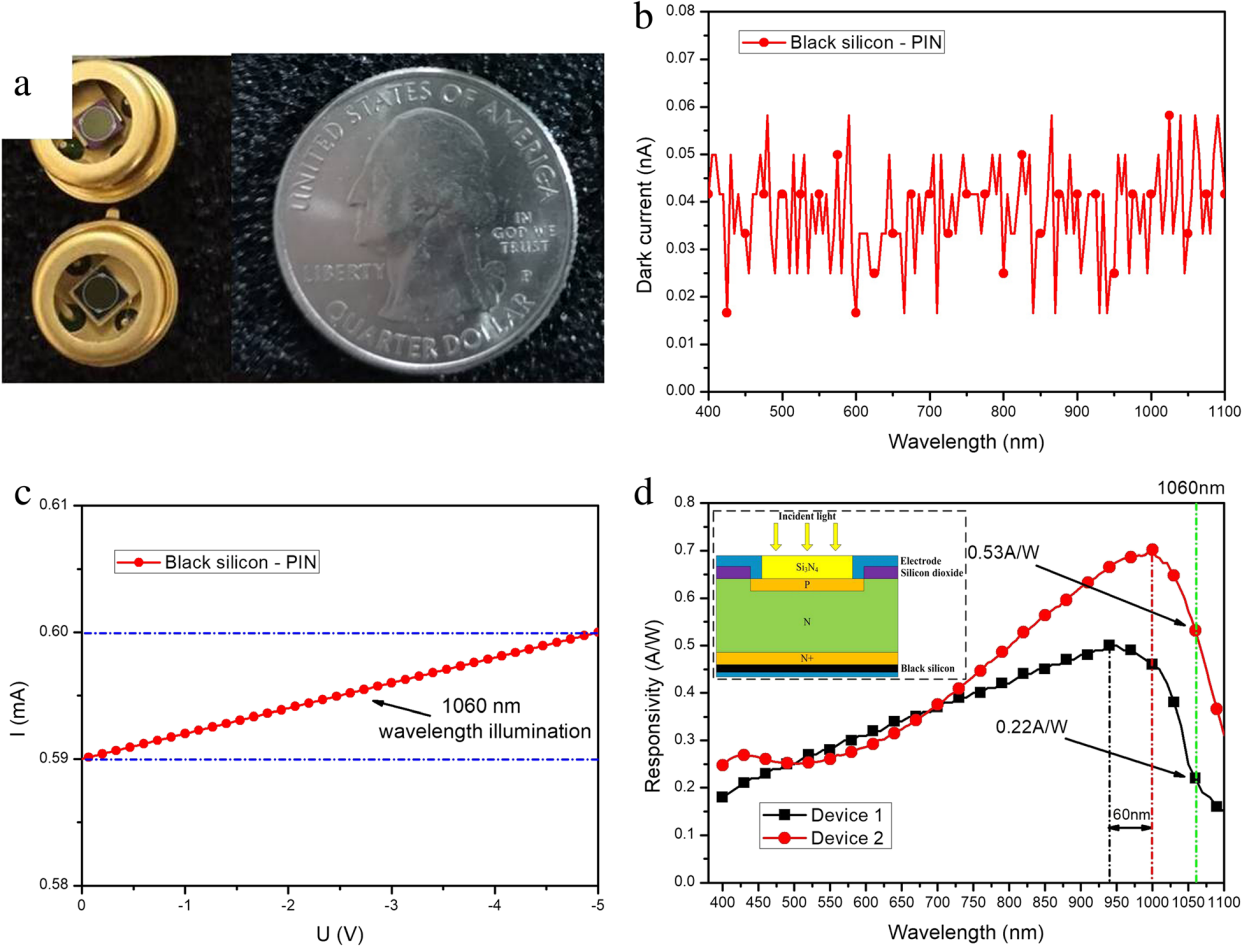
Detector image (**a**), dark current (**b**), I–V curve under 1060 nm wavelength illumination (**c**), and responsivities of two different detectors (**d**): device 1 from ref. [22] and device 2 based on the results of present paper. The inset of **d** shows the device structure

Based on the above black silicon with enhanced optical properties, a Si-PIN detector with black silicon formed on the back surface has been fabricated. First, a pure intrinsic monocrystalline silicon wafer (n-type) is oxidized on both sides forming SiO_2_ layers. Second, the P layer is fabricated by boron diffusion on the photosensitive region that is formed early by etching the SiO_2_ layer on the front surface of the wafer through photolithography process. Third, a layer of Si_3_N_4_ permeation film is deposited on the P layer, and then the back surface of the wafer is polished and grinded to about 200 μm thickness. Fourth, a P-doped N^+^ layer is deposited on the grinded surface, and then the microstructured black silicon is formed on the top of N^+^ layer. Finally, the electrode windows are etched by photolithography process and metal electrodes are deposited on both sides of the wafer. Figure 8 gives a real device image (a), dark current (b), I–V curve under 1060 nm wavelength illumination (c), and the responsivity comparison of two different detectors (d). It is hereby declared that the responsivity of device 1 (S1336-44BK, a commercial Si-PIN detector) is re-plotted based on the public Website of Hamamatsu Photonics Company [22], and the responsivity of device 2 is obtained on our newly fabricated Si-PIN detector with black silicon formed on the back surface, in which the photosensitive surface was a circle with a diameter of 2 mm. It can be clearly seen that device 2 performs a substantial increase in responsivity, particularly at near-infrared wavelengths, i.e., 0.53 A/W at 1060 nm and 0.31 A/W at 1100 nm, respectively.

It can be seen from Fig. 8b that although the Si-PIN detector with black silicon formed on the back surface (device 2) shows a relatively little improvement responsivity in visible spectrum, the response spectrum of it gives an even higher responsivity in the wavelength range from 680 to 1100 nm with about 60 nm red shift of peak responsivity, compared with the commercial Si-PIN detector (device 1). The main reason for such a distinction is that the device structure of these two detectors (devices 1 and 2) is different. When the photon energy is greater than the band gap of C-Si, the incident light is mainly absorbed by P layer and so the generated carriers have enough energy to transit N layer. Most of the generated carriers can be collected by N^+^ layer to output photocurrent through electrode. In this condition, whether the back surface of the detector is introduced with or without black silicon, there will be a limited influence on the device response in the visible wavelength. Different from the detector with black silicon at the front surface [21], device 2 demonstrates a better response in the visible wavelength. That is why there is a relatively little improvement in visible light response according to the measured responsivity curve. Again in device 2, because the black silicon layer is set on the back surface, even if the photon energy is less than the band gap of C-Si, near-infrared light is able of penetrating P layer and absorbed by N layer, and then a large number of generated carriers are able to be collected by the N^+^ layer under the action of reverse bias. As a result, there will be a countable photocurrent output and the device represents a substantial responsivity increase in the near-infrared wavelength.

According to above results, our present study could provide a feasible strategy for near-infrared photoelectronic detection field, but there are still a lot of aspects that should be considered. For example, better fabrication processes and ion implantation technologies of microstructured black silicon, which could precisely control the morphologies and band gaps of the structured silicon should be explored. Furthermore, some other novel device structures of photoelectronic detector based on black silicon should be designed in order to realize a better device performance.

## Conclusions

In summary, the microstructured black silicon materials are fabricated by two-step process: deep reactive ion etching combined with plasma immersion ion implantation. The microstructured cylindrical arrays on the surface of silicon wafers have three different sizes: mask I (*D* = 4 μm, *T* = 6 μm), mask II (*D* = 4 μm, *T* = 8 μm), and mask III (*D* = 4 μm, *T* = 10 μm), with the height of 1.87 μm, 2.35 μm, and 3.15 μm, respectively. Obviously enhanced light absorptance of black silicon has been obtained in a wide wavelength range from 400 to 2000 nm, and the maximum and average light absorptance reach 83% and 62%, respectively. These enhancements are discussed extensively based on multiple reflection, increased absorption path length, and narrow band gap. A novel Si-PIN photoelectronic detector with black silicon formed on the back surface has been fabricated, and a comparison of device responsivity has been made with one commercial device named as S1336-44BK. It is concluded that our Si-PIN photoelectronic detector with black silicon formed on the back surface has a substantial increase in responsivity, particularly in the near-infrared wavelengths, rising to 0.53 A/W at 1060 nm and 0.31 A/W at 1100 nm, respectively.
